# Cystic fibrosis and renal disease: a case report

**DOI:** 10.1186/1752-1947-1-24

**Published:** 2007-06-04

**Authors:** Baha A Al-Shawwa, Aparna R Rao

**Affiliations:** 1 Department of Pediatrics, Medical College of Wisconsin (Pulmonary Section), Children's Hospital of Wisconsin, 9000 West Wisconsin Avenue, MS # B620, Milwaukee, WI 53226, USA

## Abstract

**Background:**

Cystic fibrosis (CF) is an autosomal recessive disease that is predominantly seen in the Caucasian population and involves multiple organs. Traditionally it has been thought that the kidney is the only organ which does not seem to be generally affected by the disease although the cystic fibrosis transmembrane conductance regulator (CFTR) gene is expressed in the kidney.

**Case presentation:**

We report the case of an 11 year old boy with cystic fibrosis and nephrotic syndrome and review the literature that describes nephrotic syndrome and renal involvement in cystic fibrosis.

**Conclusion:**

With continued advances in the management of cystic fibrosis and improvement in life expectancy, several unrecognized co-morbidities are expected to emerge. It is important to screen patients for possible co-morbidities. Urine analysis may be helpful in this group of patients and any proteinuria should raise the suspicion of cystic fibrosis-related renal disease.

## Background

Cystic fibrosis (CF) is a multisystem disease characterized by chronic respiratory infections and exocrine pancreatic insufficiency. Recent advancement in therapy has lead to improvement in survival. Currently, the median age for a patient with CF is the early 30's [1]. CF is no longer only a pediatric disease and long term complications are being more frequently reported in adults.

Patients with CF usually present with symptoms involving the respiratory and gastrointestinal systems. However, other systems can be involved in CF including the renal system. Traditionally the only abnormalities associated with the renal tract are nephrolithiasis [2,3] and mechanical urological problems associated with coughing [4]. In this case report, we describe a patient with cystic fibrosis and nephrotic syndrome. We also review the literature about renal involvement in CF.

## Case presentation

An 11 year old male with CF (homozygous for ΔF508), mild lung disease and pancreatic insufficiency presented with facial swelling that progressed to generalized anasarca over four days. There was a history of preceding upper respiratory symptoms. He denied any change in his urine color or bowel habit and there was no history of headache, visual disturbance, jaundice, chest pain or palpitation. The patient remained on his regular medications which included albuterol, multivitamins and pancreatic enzymes.

He was admitted with similar symptoms 6 months prior to this admission. At that time he had hypoalbuminemia without proteinuria on a random urine sample. An extensive workup was negative including liver function tests, viral hepatitis panel, Alpha 1 antitrypsin (stool, blood), abdominal ultrasound and upper GI endoscopy. He was treated with albumin infusion and furosemide and no corticosteroids were needed. However, the etiology for the hypoalbuminemia was uncertain.

On this admission, he had mildly elevated blood pressure at 131/73. His weight was 46.7 kg with 8 kg of recent weight gain. He had periorbital and facial edema and moderate pitting edema of both lower extremities and around the sacral area. Otherwise physical examination was normal.

Laboratory evaluations showed hypoalbuminemia (1.6 mg/dl) with normal kidney function (BUN of 11 mg/dl, creatinine of 0.4 mg/dl and normal urine microscopic evaluation without evidence of RBC casts). 24 hour urine collection revealed nephrotic range proteinuria (3 gm/24 hour). Other laboratory evaluations were normal including ASO, C3, C4 and ANA. He underwent percutaneous renal biopsy which revealed minimal change disease. The interstitium showed scattered mixed mononuclear inflammation with rare eosinophils and neutrophils without any evidence of fibrosis. There was no immune deposit and no significant glomerulosclerosis. The patient was treated successfully with oral corticosteroids 2 mg/kg/day and achieved remission after about six weeks of therapy.

## Discussion

There is a general perception that the kidney is spared in patients with CF. However in recent years there have been increasing reports of renal disease in patients with CF. Several anatomical and pathological reports describe renal abnormalities in association with CF although there is still a gap in clinical reporting. Glomerular alterations including glomerulosclerosis [5], deposits of immune complexes [6], IgA nephropathy [7] and mesangial proliferations, nephrocalcinosis and microscopic hematuria [8], tubular injury [5,9], diabetic nephropathy [10], fibrillary glomerulonephritis [11] and amyloidosis [12,13] have all been described in patients with CF. There are to our knowledge, only two cases that of nephrotic syndrome related to minimal change disease [14,15] which have been reported in the medical literature.

Although nephrotic syndrome is rarely encountered with CF, mild proteinuria is not infrequently found on urine analysis [5,12]. Castile *et al*. reported that five out of 23 patients reviewed in an autopsy series had had unexplained proteinuria (range, trace to +2) recorded on routine urine analysis and the majority of these patients were noted to have renal pathology at autopsy.

## Conclusion

Nephrotic syndrome in this patient with cystic fibrosis could either be coincidental or a complication of CF. With continued advances in the management of CF and improvement in life expectancy, several unrecognized co-morbidities are expected to emerge and it is important that patients be screened for possible co-morbidities. Patients with CF are exposed to potentially nephrotoxic factors, including chronic and acute bacterial infections, with circulating immune complexes, and antibiotics especially aminoglycosides. We know that cystic fibrosis-related diabetes can produce the same microvascular complications including nephropathy recognized in the non-cystic fibrosis patient population [16-18]. Urine analysis may be helpful in this group of patients as a screening tool.

## Abbreviations

CF = Cystic fibrosis; IgA = Immunoglobulin A; RBC = Red Blood Cell.

## Competing interests

The author(s) declare that they have no competing interests.

## Authors' contributions

BA collected the data and drafted the manuscript. Both BA and AR revised and approved the final manuscript.
